# Polymicrobial Pyogenic Liver Abscess Without Classical Risk Factors: A Case Report of Streptococcus constellatus and Fusobacterium nucleatum Infection

**DOI:** 10.7759/cureus.90195

**Published:** 2025-08-15

**Authors:** Drew W Barron, Sophie G Wallace, Douglas E Rappaport, James M Kelley, Wayne A Martini

**Affiliations:** 1 Emergency Medicine, Mayo Clinic Alix School of Medicine, Phoenix, USA; 2 Emergency Medicine, University of Arizona, Phoenix, USA; 3 Emergency Medicine, Mayo Clinic, Phoenix, USA

**Keywords:** atypical presentation, broad-spectrum antibiotics, computed tomography (ct) imaging, fusobacterium nucleatum, hepatic abscess, immunocompetent host, percutaneous drainage, polymicrobial infection, pyogenic liver abscess, streptococcus constellatus

## Abstract

Pyogenic liver abscesses are uncommon, often polymicrobial, and can be fatal if left untreated, with a mortality rate of 5-15%. Established risk factors include diabetes mellitus, hepatobiliary disease, malignancy, recent abdominal surgery, and immunosuppression. This case report describes a 69-year-old female who developed a large hepatic abscess despite lacking traditional predisposing risk factors.

The patient is a 69-year-old female, generally healthy with only a history of osteopenia and hyperlipidemia, who presented with progressive fevers, chills, fatigue, myalgias, nasal congestion, minimally productive cough, decreased appetite, and a single episode of non-bloody, non-bilious vomiting. She had no abdominal pain but endorsed nausea and significant anorexia. Her laboratory evaluation revealed leukocytosis and elevated transaminases, coupled with a persistent fever, nausea, and anorexia, prompting a contrast-enhanced computed tomography (CT) of the abdomen and pelvis. The scan identified a hepatic abscess in the right hepatic lobe measuring 6.1 x 8.1 x 8.9 cm (volume = 439.75 cm³). The abscess was drained by interventional radiology, and cultures from the aspirate ultimately grew *Streptococcus constellatus *and *Fusobacterium nucleatum*; a percutaneous drain was left in place. After confirming penicillin tolerance, the patient was transitioned to oral amoxicillin-clavulanate for outpatient management. Follow-up CT imaging at two weeks showed marked improvement, enabling drain removal. Complete resolution was confirmed on ultrasound at two months.

This case highlights the importance of maintaining a broad differential diagnosis and utilizing early laboratory tests and CT imaging when systemic signs of infection are present, even in the absence of traditional risk factors for a hepatic abscess. The patient's vague gastrointestinal symptoms of anorexia and nausea, along with the absence of focal abdominal pain or jaundice, reinforce the subtle clinical manifestation in this case. A thorough approach to identifying the cause of fever in the setting of gastrointestinal symptoms is crucial for making the challenging diagnosis of hepatic abscesses. Early detection through timely laboratory studies and imaging, combined with appropriate antimicrobial and procedural interventions, was imperative for achieving a favorable outcome in this patient. As pyogenic liver abscesses continue to be reported in patients without classical risk profiles, clinicians must remain vigilant in pursuing definitive diagnosis and management strategies in similar atypical clinical scenarios.

## Introduction

Liver abscesses are relatively rare but potentially life-threatening infections, most commonly of pyogenic origin and often polymicrobial, with frequent involvement of *Streptococcus* species, *Escherichia coli*,* Klebsiella pneumoniae*, *Staphylococcus aureus*, and anaerobic bacteria [1]. If left untreated, these infections can lead to sepsis, multiorgan failure, and death [1]. Even with appropriate treatment, they remain clinically significant, with reported inpatient mortality of 7.8% and recurrence rates of 13.7%. Additionally, larger abscess size has been shown to independently predict prolonged hospitalization and worse clinical outcomes [2].

Traditional risk factors for pyogenic liver abscess include diabetes mellitus, underlying hepatobiliary disease, recent abdominal surgery, malignancy, or immunosuppression [2,3]. In this case, our patient was a previously healthy 69-year-old female without any of these predisposing conditions, making her presentation atypical. The identification of *Streptococcus constellatus* and *Fusobacterium nucleatum* as causative organisms in a large abscess in an immunocompetent host underscores the importance of maintaining a broad differential and considering hepatic abscess even in the absence of classic risk factors.

## Case presentation

We present the case of a 69-year-old female with a past medical history significant for osteopenia, hyperlipidemia, and chronic cough who developed progressively worsening illness in the two weeks leading up to her evaluation in the emergency department.

Her symptoms included progressive fevers up to 103.4 °F, chills, fatigue, myalgias, nasal congestion, and a minimally productive cough. She also noted decreased appetite and experienced a single episode of non-bloody, non-bilious vomiting. She explicitly denied abdominal pain or diarrhea. Additionally, she endorsed headaches but denied photophobia, neck stiffness, or rash.

After the onset of her symptoms, she initiated a short course of oseltamivir (Tamiflu; Roche, Basel, Switzerland) without improvement. She presented to the emergency department on day five of the Tamiflu course. Notably, she was not tested for influenza before starting the medication. Around the same time, she was evaluated at an outside urgent care, where she was told her symptoms were likely due to a urinary tract infection based on the presence of inflammatory cells in her urine. She was prescribed trimethoprim-sulfamethoxazole (Bactrim; Roche, Basel, Switzerland), also on day five of her illness. Subsequent urine cultures were negative. At the time of that evaluation at the urgent care, she tested negative for both COVID-19 and influenza.

She denied recent foreign travel, use of immunosuppressive medications, or recent surgical procedures, injections, or known exposures. She has no known underlying liver disease. Her most recent colonoscopy, performed three years prior, revealed a single 3-mm tubular adenoma.

On physical examination, the patient was alert, oriented, and non-toxic appearing. She did not appear acutely ill or uncomfortable. She exhibited no respiratory distress or labored breathing. HEENT (Head, Eyes, Ears, Nose, and Throat) examination revealed moist mucous membranes, pink conjunctivae, and anicteric sclerae. The neck was supple without lymphadenopathy, jugular venous distension, or meningismus. The cardiovascular examination revealed a regular rate and rhythm, with no murmurs, rubs, or gallops. Pulmonary auscultation revealed clear breath sounds bilaterally without wheezing or rales. Abdominal examination revealed a soft, non-distended abdomen with normal bowel sounds. There was no tenderness to palpation, rebound, guarding, organomegaly, or palpable masses. No flank tenderness was appreciated. No ascites was noted. The extremities were warm and well-perfused, with 2+ distal pulses and no edema. The patient moved all extremities spontaneously without focal deficits. The skin was warm and well-perfused, with a few scattered excoriations. Vital signs on presentation were reassuring. Her temperature was slightly elevated at 99.86 °F, heart rate 81 beats per minute, respiratory rate was elevated at 24 breaths per minute, and blood pressure 105/58 mmHg, with a calculated mean arterial pressure of 70 mmHg while supine. Oxygen saturation was 92% on room air. Body mass index was 22.4 kg/m². She reported a pain score of 0/10 at the time of evaluation.

During her emergency department course, the patient experienced intermittent hypotension, dropping as low as 86/61 mmHg, although this improved following administration of two liters of intravenous fluids.

Given the nonspecific and non-localizing symptoms observed during the history and physical examination, a broad differential diagnosis was considered, and a comprehensive diagnostic approach was implemented. Initial testing included a complete blood count (CBC), comprehensive metabolic panel (CMP), and chest radiograph (CXR). The patient was also treated supportively with two liters of intravenous fluids to address hypotension, and she was administered 50 mg of intravenous ketorolac (Toradol; Roche, Basel, Switzerland) for symptomatic relief. Laboratory testing revealed leukocytosis with a white blood cell count of 13.9 x10⁹/L, neutrophil predominance (12.07 x10⁹/L), elevated transaminases with alanine aminotransferase (ALT) of 124 U/L, and aspartate aminotransferase (AST) of 64 U/L, and elevated alkaline phosphatase at 379 U/L. Coagulation studies showed a prolonged prothrombin time (17.1 sec) and an elevated international normalized ratio (INR) of 1.5. Additional notable findings included hyponatremia (131 mmol/L), hypochloremia (92 mmol/L), and mildly decreased albumin (3.4 g/dL). These abnormalities prompted further evaluation for a systemic or hepatobiliary source of infection or inflammation. Complete initial laboratory values are summarized in Tables 1, 2, 3.

**Table 1:** Emergency Department Laboratory Results (H): High; result is above the reference range. (L): Low; result is below the reference range. Blank: Result is within the normal reference range. MCV, Mean Corpuscular Volume; RBC, Red Blood Cell; INR, International Normalized Ratio; BUN, Blood Urea Nitrogen; ALT, Alanine Aminotransferase; AST, Aspartate Aminotransferase

Test	Reference Range and Units	Result
Hemoglobin	13.2–16.6 g/dL	12.8
Hematocrit	38.3–48.6%	37.5
MCV	78.2–97.9 fL	91.9
RBC Distribution Width	11.8–14.5%	13
Platelet Count	135–317 x10⁹/L	346 (H)
Leukocytes	3.4–9.6 x10⁹/L	13.9 (H)
Neutrophils	1.56–6.45 x10⁹/L	12.07 (H)
Lymphocytes	0.95–3.07 x10⁹/L	1.16
Monocytes	0.26–0.81 x10⁹/L	0.57
Eosinophils	0.03–0.48 x10⁹/L	0.03
Basophils	0.01–0.08 x10⁹/L	0.03
Prothrombin Time (PT)	9.4–12.5 sec	17.1 (H)
INR	0.9–1.1	1.5 (H)
Sodium	135–145 mmol/L	131 (L)
Potassium	3.6–5.2 mmol/L	4.2
Chloride	98–107 mmol/L	92 (L)
Bicarbonate	22–29 mmol/L	25
Anion Gap	7–15	14
BUN	8.0–24.0 mg/dL	9.9
Creatinine	0.74–1.35 mg/dL	0.92
Calcium, total	8.8–10.2 mg/dL	9.2
Glucose	70–140 mg/dL	123
Bilirubin, Total	0.0–1.2 mg/dL	0.3
Bilirubin, Direct	0.0–0.3 mg/dL	0.2
ALT	7–55 U/L	124 (H)
AST	8–48 U/L	64 (H)
Alkaline Phosphatase	40–129 U/L	379 (H)
Protein, Total	6.3–7.9 g/dL	6.4
Albumin	3.5–5.0 g/dL	3.4 (L)

**Table 2:** Abnormal Urinalysis Indicative of Possible Lower Urinary Tract Infection With Sample Contamination HPF: high-power field; LFP: low-power field

Urinalysis Results:	Reference Range and Units	Result
Appearance	Clear, yellow	Clear, straw-colored
Glucose	Negative	Negative
Ketones	Negative	Negative
Bilirubin	Negative	Negative
Nitrites	Negative	Negative
Casts (general)	Negative	Negative
Hemoglobin	Negative	Trace
Protein	Negative to trace	1+ (Grade 1)
Urobilinogen	0.1–1.0 mg/dL	2.0 mg/dL
Leukocyte esterase	Negative	Moderate
RBCs (per HPF)	0–2/HPF	3–10/HPF
WBCs (per HPF)	0–5/HPF	4–10/HPF
Bacteria	None	Moderate
Squamous epithelial cells	Occasional	Numerous
Hyaline casts (per LPF)	0–2/LPF	7/LPF

**Table 3:** Viral PCR, Iron Studies, Vitamin Levels, and Hepatitis Serologies Indicating Iron Deficiency and Elevated Vitamin B12 With Negative Infectious Workup PCR: polymerase chain reaction

Test	Reference Range and Units	Result
SARS-CoV-2 PCR	Negative	Negative
Influenza PCR	Negative	Negative
Serum Iron	35–145 mcg/dL	17 mcg/dL
Transferrin Saturation	14–50%	9%
Total Iron-Binding Capacity	250–400 mcg/dL	186 mcg/dL
Ferritin	11–328 mcg/L	714 mcg/L
Folate	>3.0 mcg/L	15.2 mcg/L
Vitamin B12	180–914 ng/L	1187 ng/L
Hepatitis B Surface Antigen	Negative	Negative
Hepatitis B Core IgM Antibody	Negative	Negative
Hepatitis C Antibody	Non-reactive	Non-reactive

Viral polymerase chain reaction (PCR) testing for both SARS-CoV-2 and influenza was negative.

Iron studies were notable for iron deficiency, with a serum iron level of 17 mcg/dL (reference: 35-145 mcg/dL), a reduced transferrin saturation of 9% (reference: 14-50%), and a low total iron-binding capacity of 186 mcg/dL (reference: 250-400 mcg/dL). Ferritin level was elevated at 714 mcg/L (reference: 11-328 mcg/L). Folate level was normal at 15.2 mcg/L, and vitamin B12 was elevated at 1187 ng/L (reference: 180-914 ng/L), possibly reflecting supplementation or acute phase reactivity.

**Figure 1:**
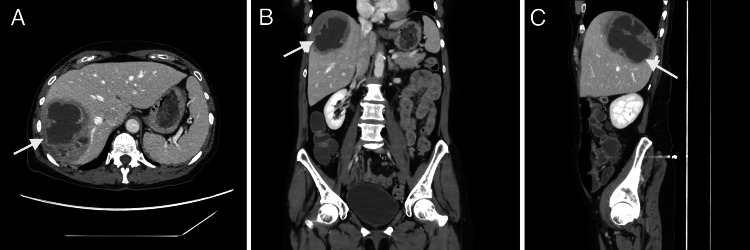
Contrast-Enhanced Computed Tomography (CT) of The Abdomen (A) Coronal (B) and sagittal (C) views of a lobulated, peripherally enhancing fluid collection within the right hepatic lobe, involving segments 5, 6, 7, and 8 (white arrow). The lesion measures approximately 6.1 x 8.1 x 8.9 cm and is surrounded by adjacent parenchymal edema, consistent with a hepatic abscess.

Viral hepatitis serologies were unremarkable. Hepatitis B surface antigen and core IgM antibody were negative, and hepatitis C antibody was non-reactive. Given the constellation of findings, including leukocytosis, elevated transaminases on laboratory results, and unexplained hypotension, blood cultures were obtained, and a computed tomography (CT) scan of the abdomen and pelvis was ordered to evaluate for an underlying source. Contrast-enhanced CT of the abdomen and pelvis, shown in Figure 1, revealed a 6.1 x 8.1 x 8.9 cm lobulated, peripherally enhancing fluid collection in the right hepatic lobe, involving segments 5, 6, 7, and 8, with surrounding parenchymal edema. These findings, in the setting of significant leukocytosis and systemic symptoms, were concerning for a hepatic abscess. There was no radiographic evidence of underlying cirrhosis, biliary dilation, or adjacent hepatic mass. A small, non-obstructing gallstone was identified without associated gallbladder wall thickening or pericholecystic fluid to suggest cholecystitis.

Following review of the imaging, interventional radiology (IR) was consulted for evaluation of image-guided percutaneous drainage and specimen collection for culture and pathology. The patient was initiated on empiric intravenous (IV) ceftriaxone 2 g in the emergency department, which she had previously tolerated without adverse reaction. She was subsequently admitted to the internal medicine service for further diagnostic evaluation, definitive management, and coordination of interventional radiology drainage. Following admission to the internal medicine service, IV metronidazole was added to broaden coverage for anaerobic organisms pending culture results. The initial plan included obtaining a transthoracic echocardiogram (TTE) to evaluate for possible endocarditis as a source of infection.

**Figure 2:**
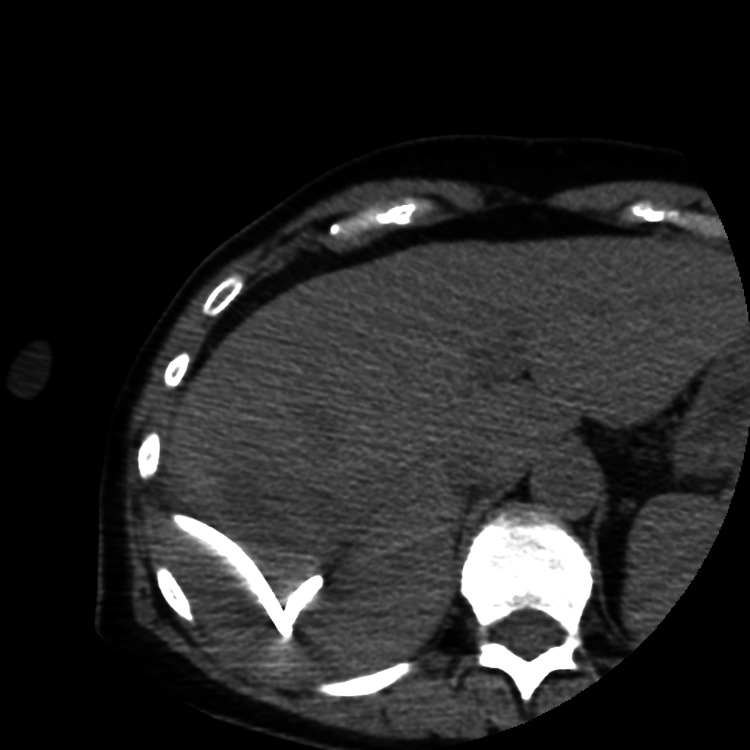
Intra-Procedural Computed Tomography (CT) Image Intra-procedural computed tomography (CT) image demonstrating placement of a 10-French drainage catheter within the hepatic abscess. The catheter is seen coiled in the fluid collection in the right hepatic lobe.

After admission, due to persistent tachypnea, a CT angiogram (CTA) of the chest was obtained to assess for pulmonary embolism, and a TTE was performed concurrently to evaluate cardiac function and rule out structural abnormalities. CTA demonstrated no evidence of acute or chronic pulmonary embolism. TTE revealed a left ventricular ejection fraction (LVEF) of 72%, normal right ventricular size and systolic function, and an estimated right ventricular systolic pressure (RVSP) of 30 mmHg. There was no evidence of hemodynamically significant valvular disease or intracardiac vegetations. The inferior vena cava (IVC) appeared normal in size with appropriate inspiratory collapse, suggesting normal right atrial pressures. On hospital day two, the IR team evaluated the patient via virtual consultation and recommended CT-guided percutaneous drainage of the hepatic abscess, with collection of fluid for bacterial and fungal cultures as well as cytologic analysis. The procedure was performed that afternoon under moderate sedation. Using intermittent CT guidance, the fluid collection in the right hemiliver was accessed, and a 10-French drainage catheter was inserted into the collection. These findings are demonstrated in Figure 2.

Fluid from the hepatic abscess was sent for bacterial, fungal, and cytologic analysis. Cultures grew *Streptococcus constellatus* (heavy growth) and *Fusobacterium nucleatum* (moderate growth). The Gram stain revealed numerous Gram-positive cocci and a few Gram-negative bacilli, consistent with the culture findings. Fungal cultures were negative after 28 days. Cytology of 7 cc of opaque pink fluid showed acute inflammation with necroinflammatory debris and no evidence of malignancy. Emergency department blood cultures remained negative after 5 days of incubation. Infectious disease (ID) was consulted following drainage and culture results. The team concluded that the findings were consistent with transient bacteremia and secondary hepatic abscess formation caused by *Streptococcus constellatus* and *Fusobacterium nucleatum*. The patient denied any recent dental procedures, and her most recent colonoscopy, performed three years prior, was unremarkable. Given the polymicrobial nature of the abscess, ceftriaxone and metronidazole were continued pending final culture data, despite *Streptococcus constellatus* typically demonstrating high susceptibility to penicillin. On hospital day three, the patient reported abdominal bloating and inability to pass gas following the drainage procedure. She was monitored clinically, and her symptoms resolved spontaneously later that day without the need for imaging or intervention.

On hospital day four, the infectious disease team was reconsulted to assess readiness for discharge and transition to oral antibiotics. Although a remote penicillin allergy was noted in her chart, the patient did not recall the reaction and believed she had tolerated amoxicillin in the past. She underwent an amoxicillin challenge (250 mg) with vital sign monitoring, which she tolerated without issue. She was subsequently transitioned to oral amoxicillin-clavulanate (875/125 mg twice daily) for a planned 14-day course, with outpatient follow-up, repeat CT imaging, and reassessment by ID to determine antibiotic cessation.

The patient was discharged in stable condition on the afternoon of hospital day five.

**Figure 3:**
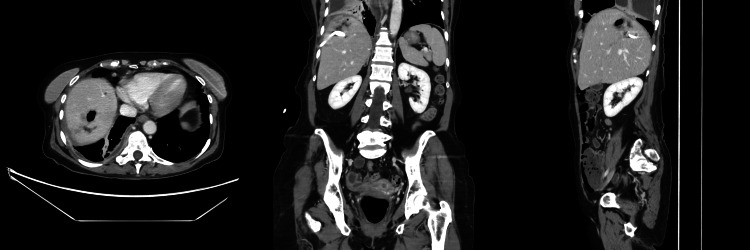
Follow-Up Computed Tomography (CT) at Two Weeks Post-Discharge. Follow-up computed tomography (CT) at two weeks post-discharge showed a reduction in abscess size within segment 8 of the liver, now measuring 3.5 cm craniocaudal (previously 8.9 cm) and 3.1 cm in axial diameter (previously 8.1 cm). The drainage catheter remained in place. No new hepatic lesions were identified. The catheter was subsequently removed without complication.

At her two-week post-discharge follow-up, repeat CT imaging, shown in Figure 3, demonstrated significant improvement, with the hepatic abscess decreasing in size to 3.5 × 3.1 cm. The drainage catheter remained in place within segment 8 of the liver and was removed by interventional radiology without complication. Despite radiologic improvement, the patient continued to experience low-grade fevers and night sweats suggestive of ongoing, though resolving, infection. Given she tolerated the initial course of amoxicillin-clavulanate well, aside from mild loose stools, the patient was prescribed an additional week of therapy. No further symptoms were reported thereafter, and it was determined that follow-up with infectious disease could proceed on an as-needed basis.

**Figure 4:**
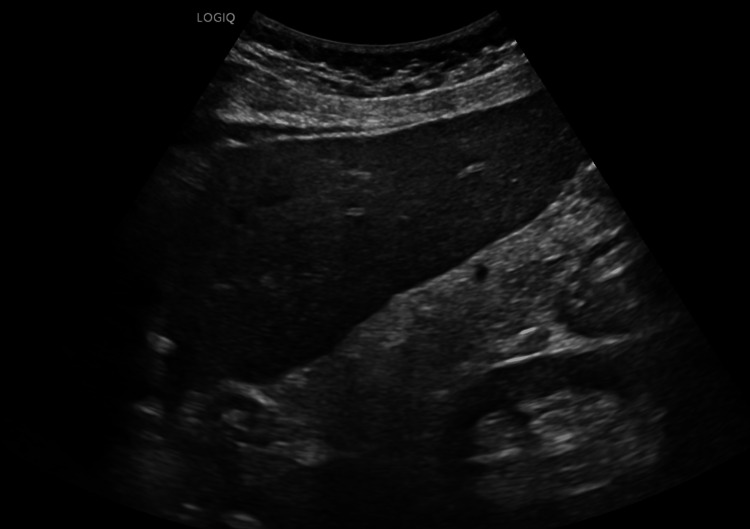
Two-Month Post-Discharge Follow-Up Liver Ultrasound. Two-month post-discharge follow-up ultrasound demonstrating a normal-appearing liver with no evidence of abscess. Mild focal fat deposition is seen along the porta hepatis.

At two months post-discharge, liver ultrasound, shown in Figure 4, demonstrated complete resolution of the hepatic abscess with normal hepatic echotexture. The only residual finding was focal fat deposition along the porta hepatis.

## Discussion

Although uncommon, pyogenic liver abscesses are increasing in incidence globally; for example, in Taiwan, the incidence rose from 10.83 to 15.45 per 100,000 person-years between 2000 and 2011 [4]. Liver infections most frequently affect individuals over the age of 50 and are more common in males [2,4]. Established risk factors include diabetes mellitus, underlying hepatobiliary disease, recent abdominal surgery or instrumentation, malignancy, and immunosuppression [1-3]. Polymicrobial infections are frequently observed, often involving *Streptococcus* species, *Escherichia coli*, *Klebsiella pneumoniae*, *Staphylococcus aureus*, and anaerobic bacteria [3].

In this case, the patient falls within the typical age range for pyogenic liver abscess but lacked nearly all traditional risk factors, including diabetes mellitus, hepatobiliary disease, malignancy, immunosuppression, or recent invasive procedures [2,4]. Her only notable past medical history included osteopenia and hyperlipidemia, and she was otherwise immunocompetent with no dental, gastrointestinal, or surgical antecedents. This highlights that a high index of clinical suspicion for liver abscess is necessary, even in patients perceived to be at low risk. The identification of *Streptococcus constellatus* and *Fusobacterium nucleatum* as causative organisms is consistent with the polymicrobial nature of many pyogenic liver abscesses, which are polymicrobial in approximately 20-50% of cases [3]. Despite historically being considered a low-virulence commensal of the oral cavity, respiratory tract, gastrointestinal tract, and urogenital system, *Streptococcus constellatus* is increasingly recognized for its pathogenic potential, particularly its role in the formation of pyogenic liver abscesses [5,6]. Similarly, *Fusobacterium nucleatum*, a commensal organism primarily found in the oral and gastrointestinal flora, has emerged as a rare but increasingly recognized pathogen in pyogenic liver abscesses, often in the absence of classic immunocompromising conditions [7].

The presence of both *Streptococcus constellatus* and *Fusobacterium nucleatum* in a large hepatic abscess in an immunocompetent patient without underlying hepatobiliary disease, malignancy, or recent procedures underscores the clinical importance of maintaining a broad differential, even in the absence of classic risk factors. Our diagnostic strategy followed a pragmatic and evidence-aligned approach, beginning with laboratory evaluation and progressing to imaging when systemic signs of infection and hepatobiliary abnormalities were identified. The patient’s leukocytosis and elevated liver enzymes prompted further evaluation via CT imaging [8,9]. Although abdominal ultrasonography is often used initially due to its lower cost and accessibility, contrast-enhanced CT is preferred for its higher sensitivity, which has been reported at 95% to 98% compared to 80% to 95% for ultrasound [10]. In this case, early use of CT imaging enabled prompt identification of the hepatic abscess. This facilitated the timely initiation of empiric antimicrobial therapy and image-guided drainage, both of which are critical for optimizing clinical outcomes. Initial management of pyogenic liver abscesses typically involves the use of empiric, broad-spectrum intravenous antibiotics targeting Gram-positive cocci, Gram-negative rods, and anaerobes [9]. In this case, the patient was started on ceftriaxone and metronidazole, a combination consistent with current recommendations for polymicrobial coverage pending culture when the causal organism is unknown [1]. Following identification of *Streptococcus constellatus* and *Fusobacterium nucleatum*, and confirmation of penicillin tolerance through a supervised oral challenge, the patient was transitioned to oral amoxicillin-clavulanate to facilitate outpatient management. This strategy aligns with literature supporting step-down oral therapy in clinically stable patients, especially when culture sensitivities are known [1].

Drain placement via image-guided percutaneous catheterization further reflects the standard-of-care for abscesses exceeding 3 cm in size or when there is clinical deterioration [1,11]. Early drainage combined with targeted antibiotic therapy has been shown to reduce recurrence and shorten hospital stays compared to antibiotics alone [11,12]. This case illustrates how early recognition, prompt imaging, image-guided drainage, and targeted antimicrobial therapy can lead to the successful resolution of a large, polymicrobial hepatic abscess. The patient’s abscess showed marked improvement at the two-week post-discharge CT scan, enabling drain removal, and was fully resolved on US at the two-month follow-up. The identification of *Streptococcus constellatus* and *Fusobacterium nucleatum*, two organisms increasingly recognized for their pathogenic potential, in a previously healthy, immunocompetent host without underlying hepatobiliary disease underscores the need to consider hepatic abscess in atypical presentations.

This case contributes to the growing body of literature demonstrating that pyogenic liver abscesses can occur in patients outside the classical risk profile, thereby reinforcing the importance of early diagnostic imaging and treatment to maximize outcomes.

## Conclusions

In this case, we presented a 69-year-old female patient with a pyogenic liver abscess caused by *Streptococcus constellatus* and *Fusobacterium nucleatum*, despite the absence of traditional risk factors such as diabetes, malignancy, hepatobiliary disease, or immunosuppression. Her presentation highlights the importance of considering hepatic abscesses in a broader spectrum of patients. As uncommon pathogens are increasingly recognized for their prominence in liver infections, clinical vigilance and a low threshold for advanced imaging and empiric therapy become critical. This case highlights the importance of proactive diagnostic approaches in ensuring early identification and favorable outcomes, even in atypical clinical scenarios.
